# Emotional, Restorative and Vitalizing Effects of Forest and Urban Environments at Four Sites in Japan

**DOI:** 10.3390/ijerph110707207

**Published:** 2014-07-15

**Authors:** Norimasa Takayama, Kalevi Korpela, Juyoung Lee, Takeshi Morikawa, Yuko Tsunetsugu, Bum-Jin Park, Qing Li, Liisa Tyrväinen, Yoshifumi Miyazaki, Takahide Kagawa

**Affiliations:** 1 Forestry and Forest Products Research Institute, 1 Matsunosato, Tsukuba, Ibaraki 305-8687, Japan; E-Mails: tmorik@ffpri.affrc.go.jp (T.M.); yukot@ffpri.affrc.go.jp (Y.T.); kagawa@ffpri.affrc.go.jp (T.K.); 2 School of Social Sciences and Humanities, University of Tampere, Kalevantie 4, Tampere FI-33104, Finland; E-Mail: Kalevi.Korpela@uta.fi; 3 Korea Forest Service, Government Complex 1, 189 Cheongsa-ro, Seo-gu, Daejeon 302-701, Korea; E-Mail: lohawi@gmail.com; 4 College of Agriculture and Life Sciences, Chungnam National University, 99 Daehak-ro, Yuseong-gu Daejeon 305-764, Korea; E-Mail: bjpark@cnu.ac.kr; 5 Department of Hygiene and Public Health, Nippon Medical School, 1-1-5 Sendagi, Bunkyo-ku, Tokyo 113-8602, Japan; E-Mail: qing-li@nms.ac.jp; 6 Finnish Forest Research Institute (METLA), PO Box 18 (Jokiniemenkuja 1), Vantaa FI-01301, Finland; E-Mail: liisa.tyrvainen@metla.fi; 7 Center for Environment, Health and Field Sciences, Chiba University, 6-2-1 Kashiwanoha, Kashiwa, Chiba 277-0882, Japan; E-Mail: ymiyazaki@faculty.chiba-u.jp

**Keywords:** forest bathing (*Shinrin-yoku*), psychological outcome, restorativeness, vitalization, emotion

## Abstract

The present study investigated the well-being effects of short-term forest walking and viewing (“forest bathing”). The hypothesis in our study was that both environment (forest *vs.* urban) and activity (walking and viewing) would influence psychological outcomes. An additional aim was to enhance basic research using several psychological methods. We conducted the experiments using 45 respondents in four areas of Japan from August to September, 2011. The hypothesis in our study was supported, because significant interaction terms between the environment and activity were confirmed regarding the Profile of Mood States (POMS) indexes, Restorative Outcome Scale (ROS) and Subjective Vitality Scale (SVS). No statistical differences between the two experimental groups in any of the ten scales were found before the experiment. However, feelings of vigor and positive effects, as well as feelings of subjective recovery and vitality were stronger in the forest environment than in the urban environment.

## 1. Introduction

Many stressors of urban life are increasingly driving humans to seek some form of stress relief [1]. In modern urbanized societies, acute and chronic stress and insufficient recovery from stress are well known as increasing problems and causes for long-term effects on health [2,3]. Stress is an important public health interest that is related to mental health problems, such as burnout syndrome, as well as cardiovascular, gastroenterological, immunological and neurological diseases [4]. This suggests that stress control is a vital issue in maintaining good health and preventing stress-related diseases in urbanized societies. Natural environments, including urban parks and natural, secondary, artificial man-made forests are generally associated with stronger positive health effects as compared to urban environments [5]. For instance, natural scenes bring higher tranquility and a reduced feeling of danger compared to urban scenes [6], while outdoor recreation in a “green” environment has been shown to relieve stress among urban inhabitants [7,8]; hence, evidence to date seems to indicate the positive health effect of a natural setting.

Several studies conducted in different countries have also reported that natural environments provide better emotional, physiological, restorative effects [9,10,11,12] and work performance [13] than urban environments. These positive effects are shown, for example, in lowered blood pressure, muscle tension and skin conductance and increased brain activity in the alpha frequency band: a change to positively-toned self-reported emotions and a recovery of attention-demanding cognitive performances after stress situations [14,15,16,17]. Furthermore, being outdoors seems to have revitalizing (positively-toned, energized state) effects [18]. Ulrich [19] reported that surgical patients assigned to rooms with windows overlooking natural scenery with trees had shorter postoperative hospital stays, received fewer negative comments in nurses’ notes and took fewer potent analgesics than those in similar rooms with windows facing a brick wall. Mitchell and Popham [20] reported a link between the amount of green space in residential areas and mortality in a population study conducted in the U.K. They found that a health gap derived from economic disparity was smaller for those living near green places. Based on the analysis of distance between residences and green space and the amount of green space, Maas *et al.* [21] reported that more green space brought better perceived general health. Conversely, from a preventive perspective, Roe and Aspinall [22] mentioned that rural walks benefited affective and cognitive restoration, especially in the poor mental health group, and highlighted the potential for green space as a method of preventing mental disorders.

The project on the therapeutic effects of forests was launched in 2004 as part of scientific and practical investigations into the health effects of forests in Japan [23]. In Japan, forests cover 68.2% of the total land area [24]. Walking in forests is a common recreational activity in Japan and is believed to promote both physical and mental health by breathing in fresh air and substances released from trees, as well as exercise and/or other healing factors associated with the forest environment [25,26]. Recent Japanese studies reported lower concentrations of cortisol, blood pressure and pulse rates, decreased sympathetic nerve activity (as measured by the low frequency/high frequency (LF/HF) component of heart-rate variability (HRV), enhanced parasympathetic nerve activity (as measured by the HF component of HRV) and higher levels of natural killer (NK) cell activity (a typical index of human immune function) in the human body when subjects walked or sat in a forest environment, as opposed to when they walked or sat in an urban environment [8,23,27,28].

With regard to the psychological responses in Japanese forest environments, the Multiple Mood Scale-Short Form (MSS) [29,30] showed higher scores for friendliness and well-being, and the MMS score of depression and State-Trait Anxiety Inventory (STAI) State Scale [31] scores were lower on days spent walking in the forest compared with the control day, especially among individuals who felt chronic mental stress [12]. Here, the reason why forests give us a health benefit can explain Miyazaki’s Nature Therapy Theory that human psychological and physiological functions have evolved to adapt to the forest environment. Therefore, forest stimulation easily facilitates psychological and physiological relaxation [32].

In a continuation of the Therapeutic Effects of Forests project, the present study was intended to investigate the well-being effects of short-term forest walking and viewing (“forest bathing”). Thus, the present study contributes to the issue by using walking and viewing in a reversed order compared to many previous studies [23,33,34]. One earlier study using walking and viewing, in that order, reported only physiological results, showing that, e.g., pulse rate and diastolic blood pressure were lower in the forest area than in the city area [28]. The hypothesis in our study is that both environment (forest *vs.* urban) and activity (walking and viewing) would influence psychological outcomes. 

An additional aim was to enhance basic research using several psychological methods. Emotional responses were previously measured by the Profile of Mood States (POMS) (e.g., [23,28,35]), MMS (e.g., [29,30]), STAI (e.g., [12]) and Zuckerman Inventory of Personal Reactions (ZIPERS) (e.g., [36]). However, other emotional measures have been seldom used. To date, restorative outcome scales have been used mainly in studies of favorite places [37]. Moreover, vitality is a new concept, which is related to restoration, but believed to be a distinct concept [18]; for which further studies comparing urban and natural settings are required. Moreover, the combination of the psychological measures in our study is novel in that an earlier study using Restorative Outcome Scale (ROS) and Subjective Vitality Scale (SVS) had the Positive and Negative Affect Schedule (PANAS), but not POMS, as an emotional measure (and used viewing before walking) [34]. A recent study with only viewing used POMS, but not other psychological measures [38]. We believe that many-sided descriptions of emotional and restorative responses might help environmental planning and management professionals develop and measure the qualities of a comfortable forest environment appropriate for forest bathing in the future.

## 2. Methods

### 2.1. Respondents

We conducted the experiments in four areas of Japan from August to September, 2011. During each experiment, to control the respondents’ dispersion by avoiding the influence of the attribute’s difference, such as age or gender, 11 or 12 male university students (45 in total) participated as respondents, none of whom reported any history of physical or psychiatric disorders (Table 1). The experiments were conducted under the regulations of the Institutional Ethical Committee of the Forestry and Forest Products Research Institute in Japan. All respondents were fully informed of the aims and procedures of this experiment, and their informed consent was obtained prior to the experiment.

**Table 1:** Summary of respondents and the day of examination.

Experimental Location	A	B	C	D
Cities, towns and villages	Nara Prefecture	Hiroshima Prefecture	Toyama Prefecture	Oita Prefecture.
	Yoshino Town	Akiota Town	Kamiichi Town	Oita City
Experiment schedule	August 3–4, 2011	August 8–9, 2011	September 6–7, 2011	September 13–14, 2011
Number of respondents	11	12	11	11
Age of respondents	21.2 ± 0.8	20.8 ± 1.5	21.4 ± 1.3	21.1 ± 1.4
Weather forest/control	Fine/Fine	Fine/Fine	Fine/Fine	Fine/Fine
Temperature average (°C) forest/control	28.4 ± 2.42/34.5 ± 2.80	26.6 ± 1.29/34.6 ± 1.44	25.2 ± 1.49/27.5 ± 0.89	28.0 ± 1.80/31.8 ± 0.88
Humidity average (%) forest/control	64.9 ± 12.9/42.6 ± 8.46	78.0 ± 6.72/56.6 ± 4.26	52.0 ± 8.46/41.5 ± 2.83	63.2 ± 7.09/59.1 ± 2.52

### 2.2. Study Sites

**Figure 1:**
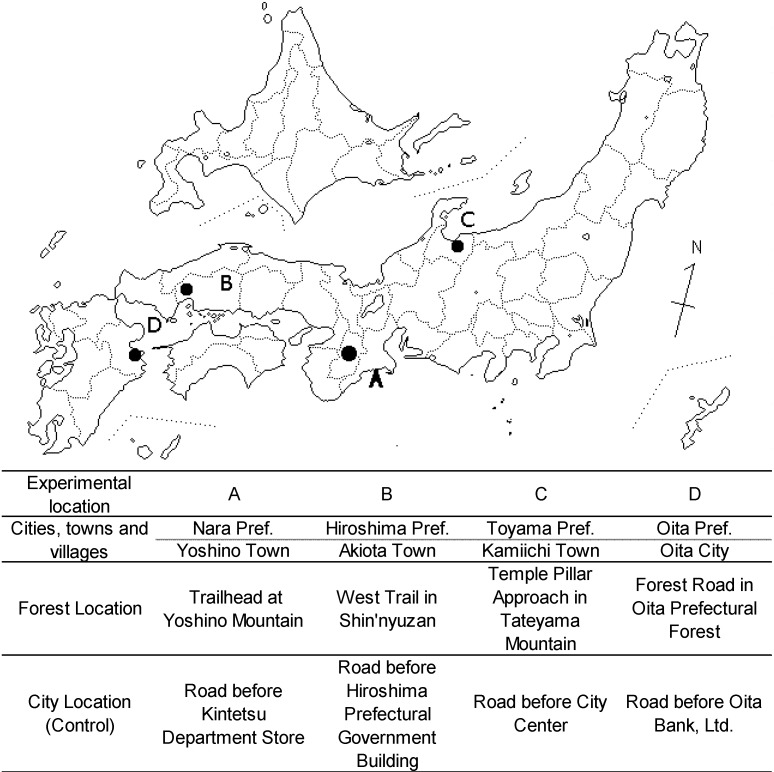
Details of each research district.

**Figure 2:**
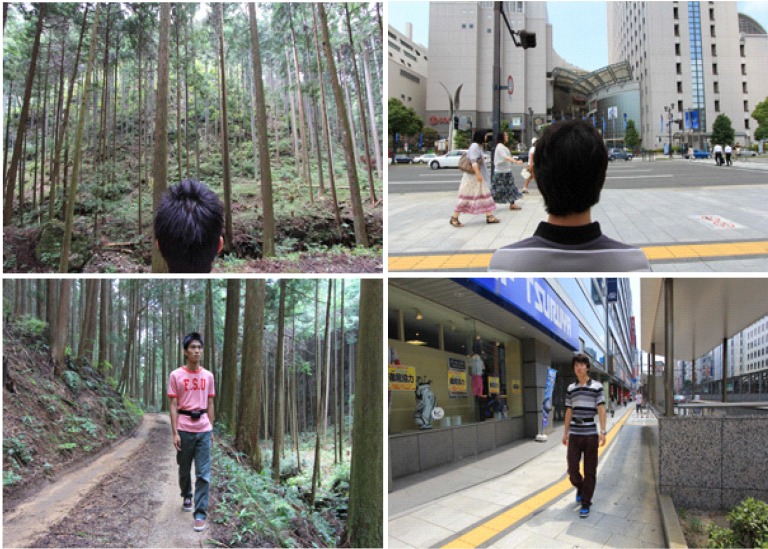
Photo showing the forest and urban site used for the Walking and Viewing sessions.

We selected four forest environments (located near the towns of Yoshino, Akiota and Kamiichi and the city of Oita) as experimental fields and four urban environments located near each of the forest environments. Figure 1 shows the location of each forest environment. The forests near Yoshino and Kamiichi are artificial, mainly comprising Japanese cedar (*Cryptomeria japonica*). The other two forests are deciduous, broad-leaved forests comprising species, such as Japanese oak (*Quercus serrata*) and Sawtooth oak (*Quercus acutissima*). The forest areas were flat, bright and well-managed (Figure 2). The urban areas were located mainly along the downtown major traffic roads or around the main station in each district. The weather was fine in each of the four research districts during the experiment. Table 1 shows the average temperature and humidity values in the forest and urban field of the four research districts. In the authors’ judgment, the outlook of the forest environments, despite the differences in tree species, was relatively similar in all research districts.

### 2.3. Psychological Measurement

Profile of Mood States (POMS): The Profile of Mood States (POMS; McNair and Lorr, 1964) is a well-established, factor-based and analytically-derived measure of psychological distress. Its reliability and validity have been well documented [39]. POMS measures six mood states: tension and anxiety (T-A), depression (D), anger and hostility (A-H), vigor (V), fatigue (F) and confusion (C). We used the Japanese version of POMS (covering 65 items) [40] and its T-score for statistical analysis.

Positive and Negative Affect Schedule (PANAS): The Positive and Negative Affect Schedule (PANAS) [41,42] measures the positive affect and negative affect using 20 items (10 each for positive and negative affect). The scales were shown to be highly internally consistent, largely uncorrelated and stable at appropriate levels over a two-month period [41]. Normative data, factorial elements and external evidence of convergent and discriminant validity for the scales are also available [41]. We used the Japanese version of PANAS (covering 16 items; 8 each for positive and negative affect) [43] and used each respondent’s total score of positive or negative affect, respectively, for statistical analysis.

Restorative Outcome Scale (ROS): The Restorative Outcome Scale (ROS) [37] is based on previous measures and findings regarding restorative outcomes [44,45,46]. ROS can be used to investigate restorative emotional and cognitive outcomes in a given environment with the following six items: “I feel calmer after being here,” “My concentration and alertness clearly increase here,” “I get new enthusiasm and energy for my everyday routines from here,” “After visiting this place, I always feel restored and relaxed,” “I can forget everyday worries here” and “Visiting here is a way of clearing and clarifying my thoughts”. Each item has a seven-point Likert scale (ranging from “1”: not at all to “7”: completely), the reliability of which was confirmed in previous research [37,47]. In this study, we used ROS after changing the original wording to suit the experiment (“1. I feel calm,” “2. I feel focused and alert,” “3. I have enthusiasm and energy for my everyday routines” “4. I feel restored and relaxed,” “5. I can forget everyday worries” and “6. My thoughts are clear.”). As there was no Japanese version available, we translated the original English version into Japanese by employing a double-check method (English into Japanese and *vice versa*). We used the total score of the six items for statistical analysis.

Subjective Vitality Scale (SVS): The Subjective Vitality Scale [48] has two versions. One assesses traits, namely the enduring characteristics of individuals, and has been found to relate positively to self-actualization and self-esteem and negatively to depression and anxiety. The other assesses the state of subjective vitality rather than its enduring aspect. At the state level, vitality has been found to relate negatively to physical pain and positively to the amount of autonomous support in a given situation. Both versions comprise seven different items, but share four common items, and a seven-point Likert scale (ranging from “1”: not at all to “7”: completely). The reliability and validity of SVS have also been confirmed in previous research [48,49]. In this experiment, we chose four common items (“I feel alive and vital,” “I don’t feel very energetic,” “I have energy and spirit” and “I look forward to each new day.”) as the SVS index, and then translated them to a Japanese version similarly to the ROS scale. We used the total score of the four items for statistical analysis (with one negative item inverted).

### 2.4. Experimental Design

#### 2.4.1. Experimental Schedule

Each experiment was conducted over two days. The respondents met at the gathering point early in the morning, then headed to the meeting room for an explanation of the field experiment and to sign a consent form. After this orientation, the respondents were randomly divided into two groups, each with five to six respondents. On the day of the experiment, depending on the area, five to six respondents (experimental group) were sent to a forest area (experimental area), while the other five to six respondents (control group) were sent to an urban area (control area). On the second day, the respondents were sent to the opposite area to eliminate order bias (Figure 1).

#### 2.4.2. Experimental Method

Upon arrival in the given area, all respondents were initially taken to see the walking course in each area by the staff before going to a pre-prepared waiting room in each area, where they were required to answer the four questionnaires in the waiting room provided in the forest environment and control areas. Subsequently, for investigating the emotional, restorative and revitalizing effects of short-term walking and viewing in forest and downtown areas, each respondent walked individually (one by one experiment; respondents were not allowed to talk to other respondents during the experiment, including the time in the waiting room) around the area during a 15-min “walking” session before noon. They also sat on chairs and viewed the scenery individually during a 15-min “viewing” session in the afternoon after a lunch break (Figure 2).

Four different questionnaires were answered at least twice during the experiment (Table 2). ROS and SVS were used three times: before the experiment, after walking and viewing and while POMS and PANAS were conducted, twice (before the experiment and after viewing).

**Table 2:** Investigative timing of each questionnaire.

	Before Experiment (B.E.)	After Walking (A.W.)	After Viewing (A.V.)
Profile of Mood States (POMS)	○	-	○
Positive and Negative Affect Schedule (PANAS)	○	-	○
Restorative Outcome Scale (ROS)	○	○	○
Subjective Vitality Scale (SVS)	○	○	○

### 2.5. Analysis Methods

In this study, we expected a significant interaction term between environment and activity. Accordingly, we set forest and urban (control) environments as factors and each measurement point (before experiment, after walking and after viewing) as levels. The interaction terms were analyzed with two-way repeated measures (within subject) ANOVA. We conducted 2 (place) × 2 (time) ANOVA for POMS and PANAS and 2 (place) × 3 (time) ANOVA for ROS and SVS for analysis.

Pairwise differences were checked with the Wilcoxon signed-rank test, and a *p*-value of <0.05 was considered significant. However, due to multiple pairwise statistical tests (Wilcoxon signed-rank test), we adjusted the *p*-value separately for each main scale with Bonferroni corrections (** *p* < 0.0017–0.005, * *p* < 0.0083–0.025). The statistical analysis of psychological data was processed using Microsoft Excel 2003 and Excel Statistics 2008 for Windows. Comparisons were made of the following pairs of situations:

POMS and PANAS: (1) responses before the experiments in both forest and control areas; (2) responses after the viewing session in both forest and urban areas; (3) responses before the experiments and after the viewing session in forest areas; and (4) responses before the experiments and after the viewing session in urban areas. 

ROS and SVS: (1) responses before the experiments in both forest and control areas; (2) responses after the walking session in both forest and urban areas; (3) responses after the viewing session in both forest and urban areas; (4) responses before the experiments and after the walking session in forest areas; (5) responses before the experiments and after the walking session in urban areas; (6) responses before the experiments and after the viewing session in forest areas; and (7) responses before the experiments and after the viewing session in urban areas.

## 3. Results and Discussion

### 3.1. Result

#### 3.1.1. Correlations of the Scales

The validity and reliability of the Japanese versions of POMS and PANAS have been confirmed in earlier studies [40,41]. However, ROS and SVS were translated to Japanese for the first time, and their convergent and discriminate validity and reliability had to be confirmed. Confirmation results of the validity and reliability are below. 

Table 3 lists the correlations of all scales before the experiment in the forest environment and control areas, while Table 4 and Table 5 show the correlations of the scales after walking and after viewing in each environment. Subsequently, Table 6 shows the result of the assessments of the reliability of scales, subscales and the whole questionnaire. 

Table 3 shows that “Negative Affect (PANAS)” is relatively high and positively correlated with negative indexes, such as tension and anxiety, depression, anger and hostility and fatigue, while relatively high and negatively correlated with such positive indexes as vigor, ROS and SVS in the forest (before the experiment) in Table 3 and Table 5. The same tendency appears for control (before the experiment) in Table 3 and Table 5. Conversely, “positive affect (PANAS)” tends to be the opposite of the “negative” index, meaning ROS and SVS have a relatively high positive correlation with the “positive” index of POMS and PANAS (Table 3 and Table 5). 

**Table 3:** Correlation of each investigative index (before experiment). T-A, tension and anxiety; D, depression; A-H, anger and hostility; V, vigor; F, fatigue; C, and confusion.

Forest (B.E.)		POMS											PANAS				ROS		SVS	
		T-A	D		A-H		V		F		C		Negative		Positive					
POMS	T-A	-	0.708	**	0.433	**	−0.101		0.575	**	0.780	**	0.430	**	0.014		−0.345	*	−0.245	
	D		-		0.623	**	0.004		0.668	**	0.646	**	0.392	**	0.006		−0.253		−0.332	*
	A-H				-		0.162		0.755	**	0.530	**	0.506	**	0.097		−0.081		−0.232	
	V						-		0.001		−0.285	*	−0.069		0.362	*	0.560	**	0.576	**
	F								-		0.617	**	0.349	*	0.092		−0.244		−0.406	**
	C										-		0.489	**	−0.029		−0.422	**	−0.402	**
PANAS	Negative												-		0.160		−0.204		−0.116	
	Positive														-		0.480	**	0.424	**
ROS																	-		0.621	**
SVS																			-	
**Control (B.E.)**		**POMS**											**PANAS**				**ROS**		**SVS**	
		**T-A**	**D**		**A-H**		**V**		**F**		**C**		**Negative**		**Positive**					
POMS	T-A	-	0.721	**	0.685	**	0.124		0.620	**	0.713	**	0.327	*	−0.010		−0.095		−0.125	
	D		-		0.815	**	0.097		0.753	**	0.654	**	0.358	*	−0.054		−0.279		−0.229	
	A-H				-		0.147		0.716	**	0.635	**	0.373	**	0.046		−0.187		−0.144	
	V						-		0.127		0.121		−0.093		0.000		0.432	**	0.415	**
	F								-		0.531	**	0.293	*	−0.082		−0.373	**	−0.473	**
	C										-		0.248		0.010		−0.152		−0.063	
PANAS	Negative												-		0.458	**	0.010		0.015	
	Positive														-		0.134		0.242	
ROS																	-		0.735	**
SVS																			-	

* *p* < 0.05; ** *p* < 0.01.

**Table 4:** Correlations between restoration and vitality scales (after walking).

Forest (A.W.)	ROS	SVS	
ROS	-	0.848	**
SVS		-	
Control (A.W.)	ROS	SVS	
ROS	-	0.856	**
SVS		-	

* *p* < 0.05; ** *p* < 0.01.

**Table 5:** Correlation of each investigative index (after viewing).

Forest (A.V.)		POMS											PANAS				ROS		SVS	
		T-A	D		A-H		V		F		C		Negative		Positive					
POMS	T-A	-	0.796	**	0.745	**	−0.095		0.630	**	0.715	**	0.542	**	0.006		−0.251		−0.144	
	D		-		0.769	**	−0.002		0.648	**	0.700	**	0.481	**	0.019		−0.284		−0.122	
	A-H				-		0.022		0.714	**	0.642	**	0.632	**	−0.076		−0.332	*	−0.272	
	V						-		0.081		−0.244		−0.224		0.449	**	0.508	**	0.444	**
	F								-		0.660	**	0.475	**	−0.083		−0.313	*	−0.420	**
	C										-		0.507	**	−0.161		−0.416	**	−0.283	
PANAS	Negative												-		−0.126		−0.356	*	−0.281	
	Positive														-		0.307	*	0.493	**
ROS																-		0.775	**	
SVS																		-		
**Control (A.V.)**		**POMS**											**PANAS**				**ROS**		**SVS**	
		**T-A**	**D**		**A-H**		**V**		**F**		**C**		**Negative**		**Positive**					
POMS	T-A	-	0.711	**	0.584	**	−0.078		0.400	**	0.504	**	0.524	**	0.035		−0.307	*	−0.143	
	D		-		0.667	**	0.021		0.415	**	0.643	**	0.493	**	−0.055		−0.244		−0.136	
	A-H				-		0.044		0.537	**	0.429	**	0.553	**	−0.054		−0.210		−0.127	
	V						-		−0.106		−0.159		−0.041		0.122		0.233		0.389	**
	F								-		0.417	**	0.267		−0.129		−0.321	*	−0.475	**
	C										-		0.487	**	−0.149		−0.214		−0.200	
PANAS	Negative												-		0.182		−0.314	*	−0.095	
	Positive														-		0.156		0.338	*
ROS																-		0.743	**	
SVS																		-		

* *p* < 0.05; ** *p* < 0.01.

**Table 6:** Verification of internal consistency.

Scales and Subscales		Cronbach’s α	
		(Sub) Scales	Whole
POMS	T-A	0.810	0.946
	D	0.894	
	A-H	0.900	
	V	0.904	
	F	0.935	
	C	0.727	
PANAS	Negative	0.889	0.875
	Positive	0.916	
ROS		0.711	0.711
SVS		0.737	0.737

ROS correlated significantly with a feeling of vigor (POMS, 0.56, *p* < 0.01) and other positive feelings (PANAS, 0.48, *p* < 0.01) before the experiment (Table 3), but also with a feeling of vigor (0.58, *p* < 0.01) and positive feelings (0.31, *p* < 0.05) after viewing (Table 5). Before the experiment, SVS (Table 3) also showed a highly significant correlation with a feeling of vigor (0.58, *p* < 0.01) and positive feelings (0.42, *p* < 0.01), as well as a feeling of vigor (0.44, *p* < 0.01) and positive feelings (0.49, *p* < 0.05) after viewing (Table 5). These results generally agreed with those obtained in the control areas. All of these correlations are less than or equal to 0.58 (explaining less than 34% of each other’s variance), suggesting that they record different concepts or psychological phenomena. However, ROS and SVS correlated significantly with each other and had even over a 50% overlap in variance before the experiment (0.62–0.74, *p* < 0.01; Table 3), after walking (0.85–0.86, *p* < 0.01; Table 4) and after viewing (0.74–0.78, *p* < 0.01; Table 5) in both environments. 

Table 6 shows the internal consistencies (Cronbach’s alphas) of all scales and subscales. The results show that POMS and PANAS have high internal consistencies, while ROS and SVS have reasonably good internal consistencies. Thus, all four measures had acceptable validity and reliability for the current experiment. 

#### 3.1.2. Interaction Terms of Environment and Activity

Interaction terms (environment and activity) were significant (*p* < 0.05) regarding POMS (tension and anxiety, vigor, fatigue, confusion), ROS and SVS (Table 7).

**Table 7:** Interaction terms by two-way repeated measures ANOVA.

Scales and Subscales		*p*-Value	Significance of Mutual Relations (Place × Time)
POMS	T-A	0.018	*
	D	0.113	-
	A-H	0.137	-
	V	0.012	*
	F	0.016	*
	C	0.011	*
PANAS	Negative	0.197	-
	Positive	0.399	-
ROS		0.020	*
SVS		0.035	*

* *p* < 0.05; ** *p* < 0.01.

Note that ROS and SVS were each measured three times during the experiment, unlike POMS and PANAS, which were measured twice (before the experiment and after the viewing activity). Regarding ROS and SVS, there are two possible interpretations of the interaction term. One is that walking activity could elicit a greater effective benefit than viewing in the forest environment. Another interpretation is that the forest provides different benefits from the city after the combined effect of walking and viewing in comparison to the before situation. Our results, except the result of SVS in the forest, support the latter interpretation. Thus, the hypothesis of this and past studies (namely, that engaging in walking and viewing activities in a natural environment, such as a forest, which is fairly different from a daily living environment, e.g., urban, would bring us greater psychological benefits) was supported.

#### 3.1.3. Comparisons between the Forest and Urban Environment

**Figure 3:**
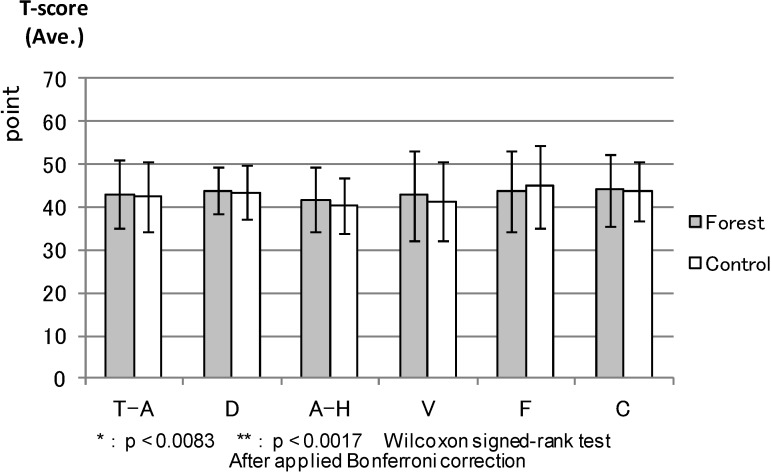
The result of POMS (before experiment).

**Figure 4:**
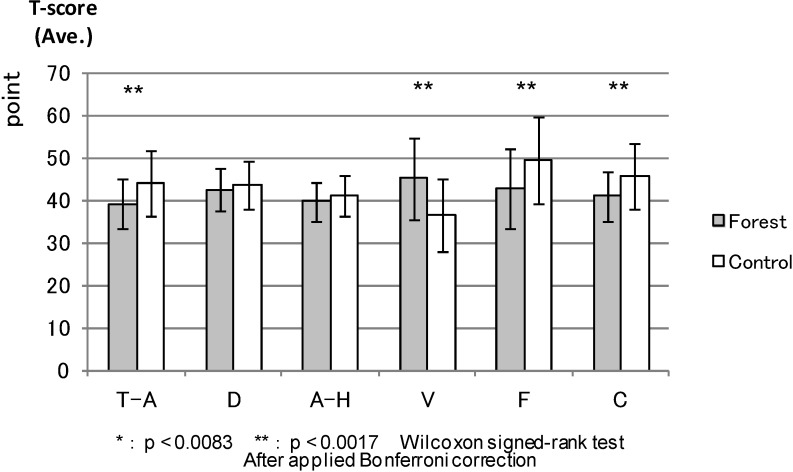
The result of POMS (after viewing).

POMS: Figure 3 compares the T-score results of the six POMS scales between the forest environment and urban areas before the experiment. There were no statistical differences. Both environments had almost the same score before the experiment. Four scales (except anger and hostility and depression) showed statistical differences between the environments after the combined effect of walking and viewing (Figure 4). More specifically, tension and anxiety (*p* < 0.000), fatigue (0.000) and confusion (0.000) were significantly lower in the forest environment than in the urban areas, whereas vigor (0.000) was significantly higher in the forest environment after the viewing session. 

**Figure 5:**
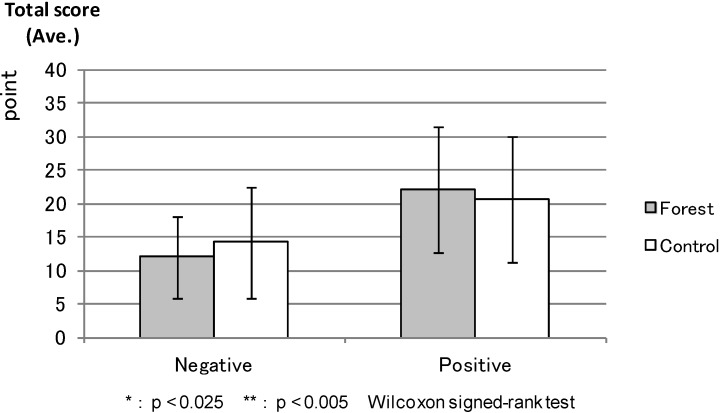
The result of PANAS (before experiment).

**Figure 6:**
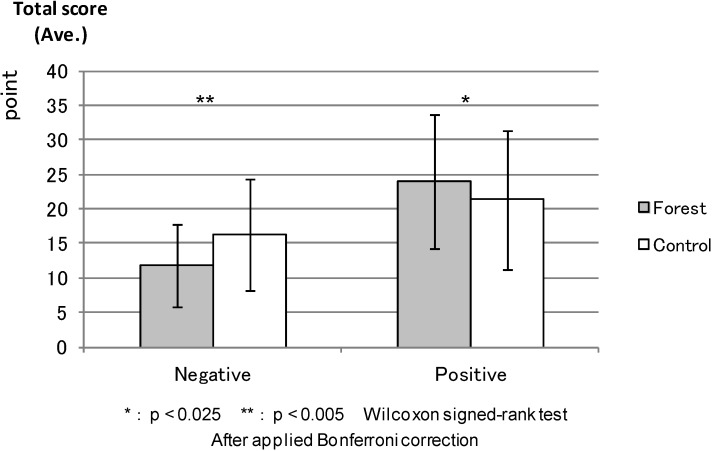
The result of PANAS (after viewing).

PANAS: Figure 5 shows the average total scores of the “Negative Affect” and “Positive Affect” scales (PANAS) before the experiment; “Negative Affect” was apparently lower and “Positive Affect” was higher in the forest environment than in the control areas, but the differences were not statistically significant. Figure 6 shows the “Negative Affect” as being significantly lower (*p* < 0.000) in the forest environment than in the urban areas, whereas the “Positive Affect” was significantly higher (*p* < 0.011) in the forest environment than in the urban areas after the combined effect of walking and viewing.

**Figure 7:**
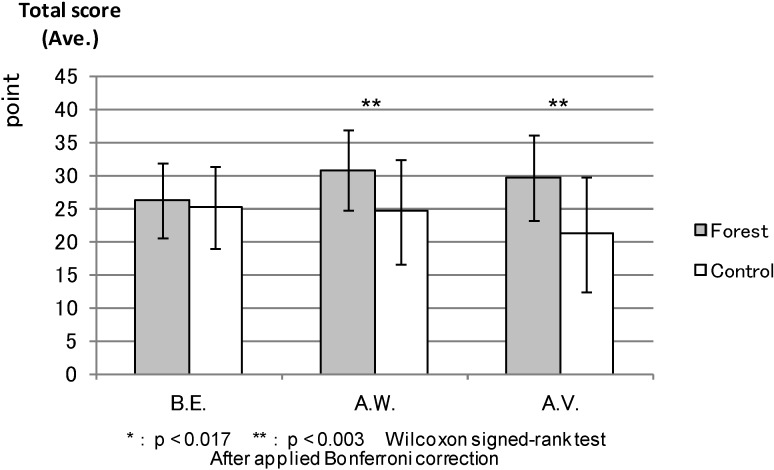
The result of ROS (forest-control).

ROS: Figure 7 compares the total ROS scores between the forest environment and the urban areas. After walking and the combined effect of walking and viewing, the ROS score was significantly higher (*p* < 0.000) in the forest environment than in the urban areas, while being almost the same in both environments before the experiment.

**Figure 8:**
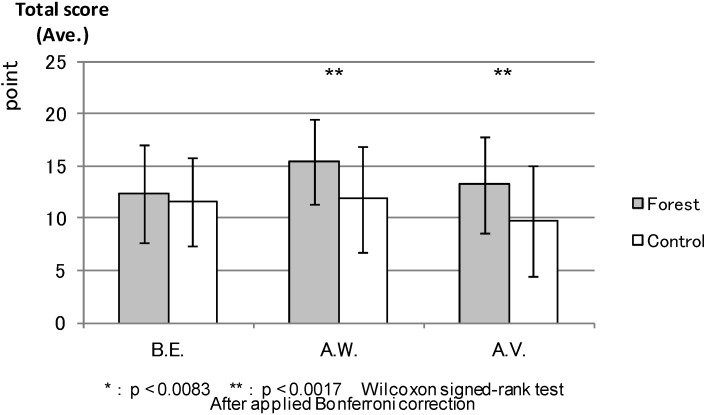
The result of SVS (forest-control).

SVS: Figure 8 compares the total SVS scores between the forest environment and the control areas. The result was relatively similar to the ROS result that showed no statistical difference between the environments before the experiment. However, the total SVS score was significantly higher in the forest environment after walking (*p* < 0.000) and after the combined effect of walking and viewing (*p* < 0.000). 

#### 3.1.4. Comparisons between before the Experiment and after Viewing and Walking

**Figure 9:**
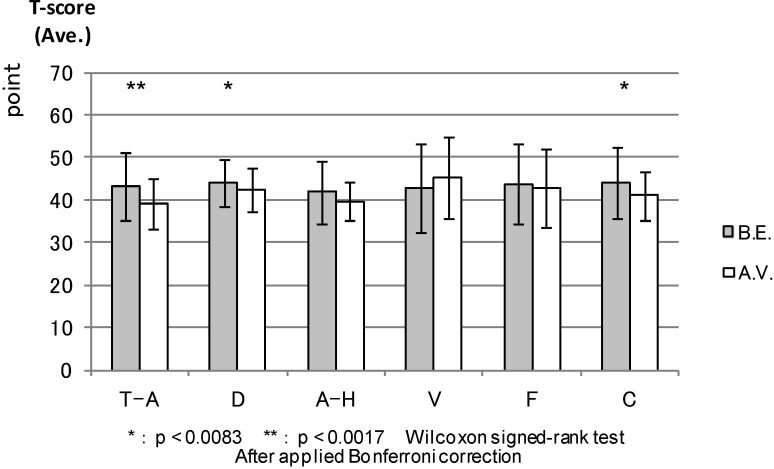
The result of POMS (forest).

**Figure 10:**
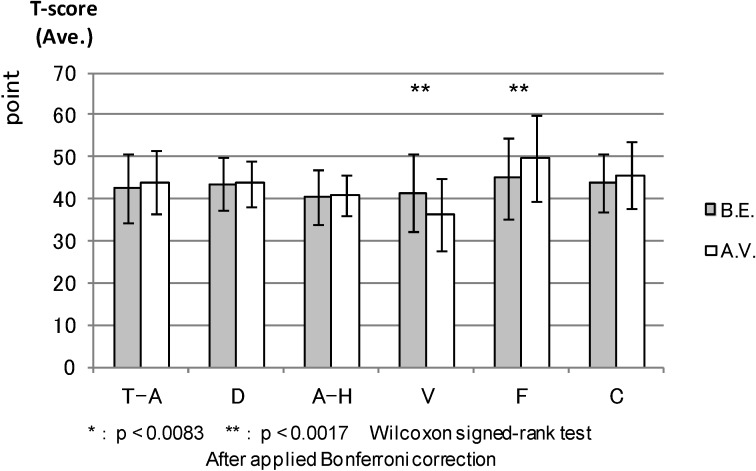
The result of POMS (control).

POMS: We compared the T-scores of all six POMS scales before the experiment and after the combined effect of walking and viewing session. Figure 9 shows that the T-scores after the combined effect of walking and viewing in tension and anxiety (*p* < 0.000), depression (0.005) and confusion (0.003) were statistically lower than those before the experiment in the forest environment. In contrast with this result, the T-score after the combined effect of walking and viewing of vigor was significantly lower (*p* < 0.000) and fatigue higher (0.000) than those before the experiment in the urban areas (Figure 10).

**Figure 11:**
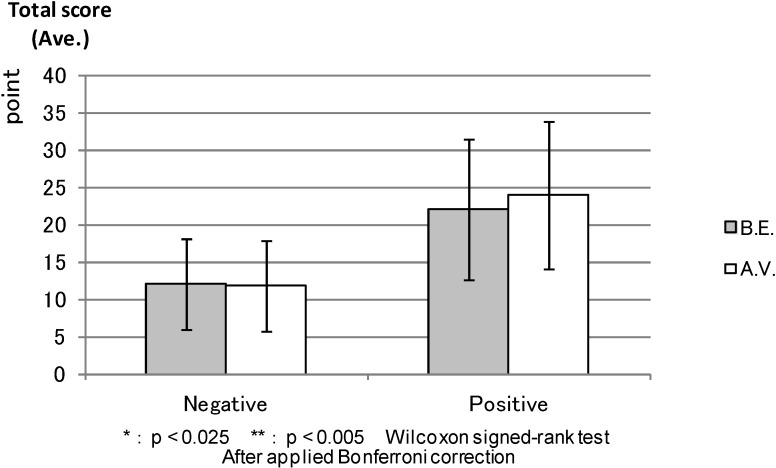
The result of PANAS (forest).

**Figure 12:**
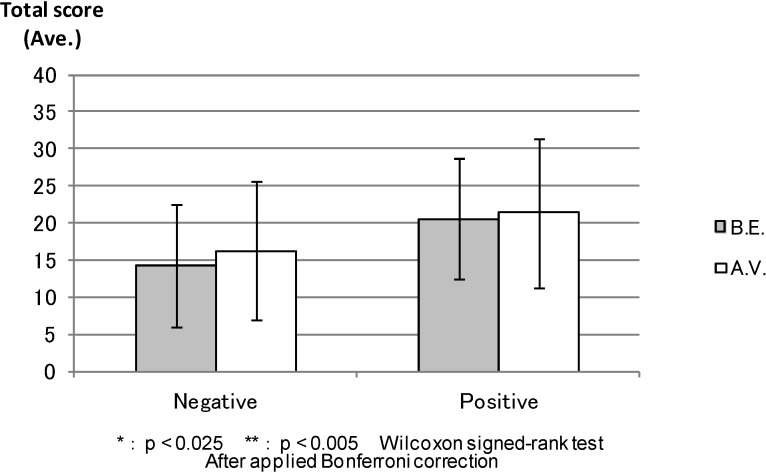
The result of PANAS (control).

PANAS: The total PANAS scores before the experiment were compared after the combined effect of walking and viewing in each environment. In Figure 11, both before the experiment and after the combined effect of walking and viewing in “Negative Affect” showed similar low scores (*p* < 0.871). Conversely, the score for after viewing is as high as that for before the experiment in the forest environment in “Positive Affect” (0.027), even though we cannot find a statistical difference between before the experiment and after viewing in either scales. Figure 12 shows that there was no statistical difference between before the experiment and after viewing either in the “Negative Affect” (*p* < 0.039) or “Positive Affect” (0.556). The “Positive Affect” score for before the experiment was also as high as that for after viewing in the urban areas. 

**Figure 13:**
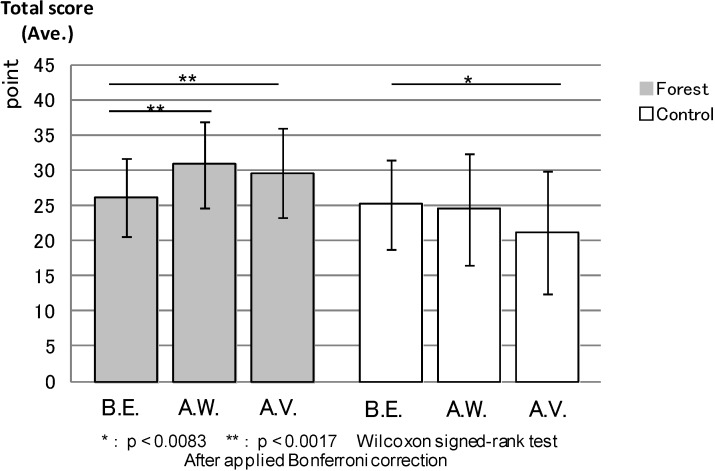
The result of ROS (forest and control).

ROS: Figure 13 shows the result of comparison among the three measurement points (before the experiment, after walking and after the combined effect of walking and viewing) in the forest environment and the urban areas. The total ROS scores for after walking (*p* < 0.000) and after the combined effect of walking and viewing (0.001) were very similar in each environment and significantly higher than before the experiment in the forest environment. In the urban areas, the score for after the combined effect of walking and viewing was relatively lower than those for before the experiment and after walking, with statistical differences between before the experiment and after the combined effect of walking and viewing (*p* < 0.002). The scores after walking (*p* < 0.830) were not statistically different from the values before the experiment.

**Figure 14:**
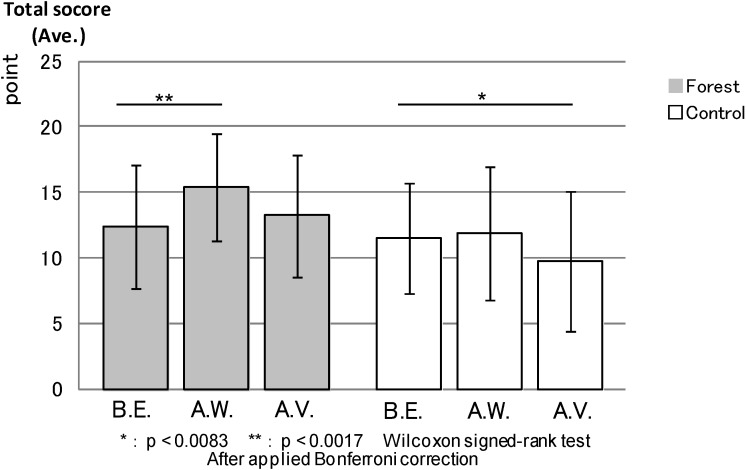
The result of SVS (forest and control).

SVS: Lastly, Figure 14 shows that, in the forest environment, the total SVS score, before the experiment was not statistically different from that for after the combined effect of walking and viewing, but after walking had statistically higher scores (*p* < 0.000) than before the experiment. Conversely, the score for after the combined effect of walking and viewing was significantly lower (*p* < 0.003) than that for before the experiment in the urban areas. There was no statistical difference between before the experiment and after walking in urban areas.

### 3.2. Discussion

#### 3.2.1. Comparisons between Forest and Urban Environment

To begin with, we found no statistical differences in the respondents’ psychological responses (mood-POMS, affect-PANAS, restorative outcomes-ROS, subjective vitality-SVS) between the forest environment and the control areas before the experiment. After a 15-min walking followed by a 15-min viewing in the both environments, we found that tension and anxiety, depression and confusion (POMS) were significantly lower, but vigor was significantly higher in the forest environment than in an urban environment. The negative affect (PANAS) was significantly lower, but the positive affect was significantly higher after a stay (the combined effect of walking and viewing) in the forest than after that in the urban environment. Scores for the emotional and cognitive restorative outcomes (ROS) were also significantly higher in the forest environment as opposed to an urban environment during (and after) the walking session, with the same results seen during (and after) the combined effect of the walking and viewing session. A short stay in the forest environment showed a significantly higher score in terms of subjective vitality (SVS) than the urban environment for both the morning (walking) and afternoon (the combined effect of walking and viewing) sessions. 

Thus, it seems that even short visits to the forest environment generate multiple positive psychological effects in mood and affect subjective recovery and vitality compared to visits to a downtown urban environment. Our main results support earlier results [18,23,25,26,27,28,35]. We did not measure any environmental factors that might induce such effects, but previous Japanese studies have highlighted coolness in a thermal environment and certain visual, audible and odorous stimuli derived from the natural setting as crucial and apparently inducing psychological recovery effects not possible in urban environments [33].

#### 3.2.2. Comparisons between before and after the Experiment

We discovered that mood worsened, negative feelings increased and feelings of restoration and vitality decreased in an urban environment. Conversely, positive feelings, subjective restoration and vitality increased and negative feelings, such as tension and anxiety, depression and confusion, declined in the forest environment after the experimental sessions. 

These results again support previous studies [23]. Both walking and the combined effect of walking and viewing during a short stay in a forest clearly induced a feeling of subjective restoration (ROS). Conversely, there was a decrease in ROS scores after the combined effect of walking and viewing in an urban environment compared to the pre-experiment score. Possible causes include heavy crowding and/or many factors derived from the urban environment, which would adversely affect respondents’ feelings of subjective restoration. 

We found a significant increase in the feeling of subjective vitality (SVS) only after the walking activity in the forest, while the subjective vitality after the combined effect of walking in and viewing an urban environment declined compared to the pre-experiment score. 

During this experiment, the walking pace in both environments was deliberately restricted. Accordingly, the physical activity itself (walking) does not explain the differences observed in subjective well-being between the forest and urban environments. Rather, these derive from the physical environment in which the activity takes place.

## 4. Conclusions

In this study, we investigated the emotional, restorative and revitalizing effects of short-term walking and viewing in forest and downtown areas. 

Initially, the hypothesis in our study that both environment and activity would influence psychological effects could be supported, because significant interaction terms between the environment and activity were confirmed regarding the POMS indexes, ROS and SVS. 

Although the subjects were once introduced to the walking course before the experiment, no statistical differences between the two experimental groups in any of the ten scales were found before the experiment. However, feelings of vigor and positive effects, as well as feelings of subjective recovery and vitality were stronger in the forest environment (after the combined effect of walking and viewing) than in the urban environment (the same timing as forest environments). 

The limitations of the present study include the fact that it was focused on the psychological effects just after the experiment, excluding potential long-term effects. This remains a topic for future studies. The experiments were carried out in flat, bright and well-managed forest areas. However, the psychological effects of forest settings may depend on the state of vegetation in the forest, as well as forest management and care. Comparisons between different kinds of forests are thus warranted in future studies. Furthermore, studies comparing different kinds of urban areas are also warranted. Methodologically, the scales of restorative outcomes and subjective vitality seemed to record somewhat similar phenomena. This applied even more so after the respondents had been exposed to the experimental manipulation (a forest or urban setting; the correlations were lower before the experiment). We need further studies to confirm whether this is due to the respondents’ positive attitude toward the experiment, to some true experimental effects or to convergence between the concepts of restoration and vitality. 

In summary, compared to the urban control, this study clarified the following psychological recovery effects derived from a short stay in the forest environment:

- Forest bathing improved mood;
- Forest bathing heightened positive affect;
- Forest bathing induced a feeling of subjective restoration;
- Forest bathing induced a feeling of subjective vitality.
